# ﻿A new freshwater species *Achnanthidiumkangdingnese* (Bacillariophyta, Achnanthidiaceae) from Sichuan Province, China

**DOI:** 10.3897/phytokeys.204.89690

**Published:** 2022-08-12

**Authors:** Pan Yu, Qingmin You, Yonghong Bi, Quanxi Wang

**Affiliations:** 1 College of Life Sciences, Shanghai Normal University, Shanghai 200234, China Shanghai Normal University Shanghai China; 2 Institute of Hydrobiology, Chinese Academy of Sciences, Wuhan, 430072, China Institute of Hydrobiology, Chinese Academy of Sciences Wuhan China

**Keywords:** Diatom, morphology, Mugecuo Lake, new species, taxonomy

## Abstract

A new freshwater diatom species, *Achnanthidiumkangdingnese* Yu, You & Wang, **sp. nov.** from Sichuan Province, China, is described. The morphology of this species was analyzed with scanning electron microscopy (SEM) and light microscopy (LM). *A.kangdingnese* belongs to the *A.initium*-like subgroup, which has external distal raphe ends curved in opposite directions of the valve. The main characteristics of *A.kangdingnese* are its linear shape, rounded apices and transpically-elongated areolae on the both valves. The central area is well defined with one or two spaced striae of the raphe valve. And on the internal valve, areolae are occluded by hymens perforated by delicate slits, and each hymen is closely joined with the adjacent hymen. We compared the new species with other similar species of *Achnanthidium*, *A.kangdingnese* is considered to be sufficiently different from other similar species based on valve outline, shape of the axial and center areas, and striae density. The new species is known only from its type locality, a mountain lake in Kangding County.

## ﻿Introduction

The genus *Achnanthidium* Kützing was initially described by Kützing (1844) as a subgenus of *Achnanthes*Bory de Saint-Vincent (1822), with *A.microcephalum* Kützing (Kützing 1844) as the type of species (Pérès et al. 2014). *Achnanthidium* was re-established by Round et al. (1990) and redefined by Round and Bukhtiyarova (1996). The number of species in *Achnanthidium* now exceeds 200 (Kociolek et al. 2018; You et al. 2021; Yu et al. 2022). Based on the characteristics of distal raphe ends, and valve and areolar shapes, the species of this genus have been divided into three major subgroups. The species of the *A.minutissimum* complex have straight external distal raphe ends and linear to linear-lanceolate valve shapes. Species in the *A.pyrenaicum* complex have external distal raphe ends that are deflected to one side and slit-like areolar openings. Members of the *A.exiguum* complex have external distal raphe ends curved in opposite directions (Yu et al. 2018, 2019a; Miao et al. 2020; Tseplik et al. 2021; Yu et al. 2022). *A.exiguum* and its relatives have since been segregated into the genus of *Gogorevia* Kulikovskiy, Glushchenko, Maltsev and Kociolek (Kulikovskiy et al. 2020). Karthick et al. (2017) also proposed the *A.initium*-like subgroup based on the external distal raphe ends which curve in opposite directions. At present, only four species belong to this latter group, including *A.contrarea* (Lange-Bertalot and Steindorf) H. Lange-Bertalot (Moser et al. 1998), *A.peridotiticum* (Moser, Lange-Bertalot and Metzeltin) H. Lange-Bertalot (Moser et al. 1998), *A.indicatrix* (Lange-Bertalot and Steindorf) H. Lange-Bertalot (Moser et al. 1998), and *A.initium* Karthick, J.C. Taylor and P.B. Hamilton (Karthick et al. 2017).

Members of *Achnanthidium* have long been considered to belong to the family Achnanthaceae. In China, 48 species of *Achnanthidium* have been reported compared to 155 taxa of *Achnanthes* (Liu et al. 2021), including 11 new *Achnanthes* species (Hustedt 1922; Jao 1964; Jao et al. 1974; Qi and Xie 1984; Zhu and Chen 1994, 1996; Kociolek et al. 2020; Liu et al. 2021). It is possible these species could belong to *Achnanthidium*, but the lack of the type material makes it difficult to confirm their taxonomic position. It is therefore necessary to collect samples from the type locality for taxonomic clarification. From 2001 to 2022, 17 new *Achnanthidium* species were described from China (Liu et al. 2016; Yu et al. 2018, 2019a, b, 2022; You et al. 2019, 2021; Liu et al. 2021; Ge et al. 2022). In the present study, we described a new freshwater diatom species, *Achnanthidiumkangdingnese* from Mugecuo Lake in Kangding County, Sichuan Province, China. We documented its valve morphology with a light microscope (LM) and scanning electron microscopy (SEM), and compared its morphological characters with similar species.

## ﻿Materials and methods

Four diatom samples were collected from Mugecuo Lake in August, 2015. The new species was only found in one sample (MGC201508036) (30°08'43"N, 101°51'35"E). Mugecuo Lake is located at an altitude of 3780 m in Kangding County, Sichuan Province, China in the northern Hengduan Mountains between the Sichuan Basin and the Qinghai-Tibet Plateau (Chen et al. 2013). Several water chemistry characteristics were also recorded, including: pH, temperature and conductivity. These were all measured using a YSIPro Plus multiparameter meter (YSI, Ohio, USA). Diatom samples were collected from natural substrates by brushing them off with clean toothbrushes. Samples were placed in sample bottles and preserved with formalin (4% final concentration).

In the laboratory, diatom samples (10 mL) were cleaned with concentrated nitric acid (10 mL) using the Microwave Accelerated Reaction System (Model MARS, CEM Corporation, Charlotte, USA) (Parr et al. 2004), with a pre-programmed digestion scheme (temperature, 180 °C) (Yu et al. 2019a). Next, samples were alternately centrifuged for 8 min at 3000 rpm (TDZ5-WS, Luyi Corporation, Shanghai, China) and washed five times using distilled water. The resulting diatom samples were preserved with 95% ethanol. Permanent diatom slides were made with Naphrax (Brunel Microscopes Ltd, Chippenham Wiltshire, U.K) for light microscopy (LM), and the cleaned diatom samples were air-dried onto cover slips and mounted onto alloy stubs for observation with the scanning electron microscope (SEM). LM studies were made with a ZEISS AXIO Imager A2 microscope fitted with DIC optics and at 1000× magnification (1.4 numerical aperture). SEM examination was made using a SU8010 (Hitachi High-Technologies Corp., Tokyo, Japan) at 2 kV, and at a working distance of less than 6 mm. Images were compiled with Adobe Photoshop CS6 (Adobe Systems Inc., San Jose, C.A., U.S.A.). Morphological terminology follows Round et al. (1990). All of the diatom samples and permanent slides are housed in the Biology Department Diatom Herbarium, Shanghai Normal University (SHTU).

## ﻿Results

### ﻿ Achnanthidium kangdingnese

Taxon classificationPlantaeCocconeidalesAchnanthidiaceae

P. Yu, Q.M. You & Q.X. Wang sp. nov.

48CB6B8D-B057-5D41-813D-25C10FF686C3

**Figure 1.**
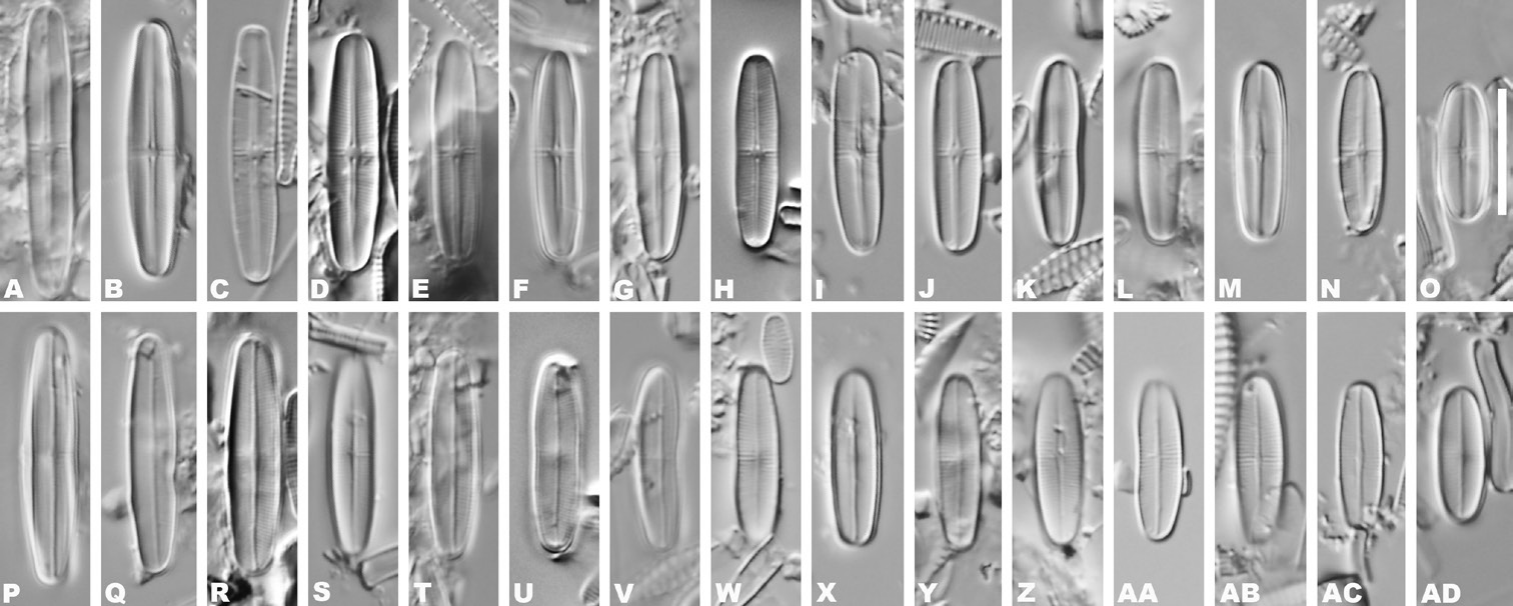
**A–AD**LM valve views of *Achnanthidiumkangdingnese* sp. nov. Scale bar: 10 µm.

**Figure 2.**
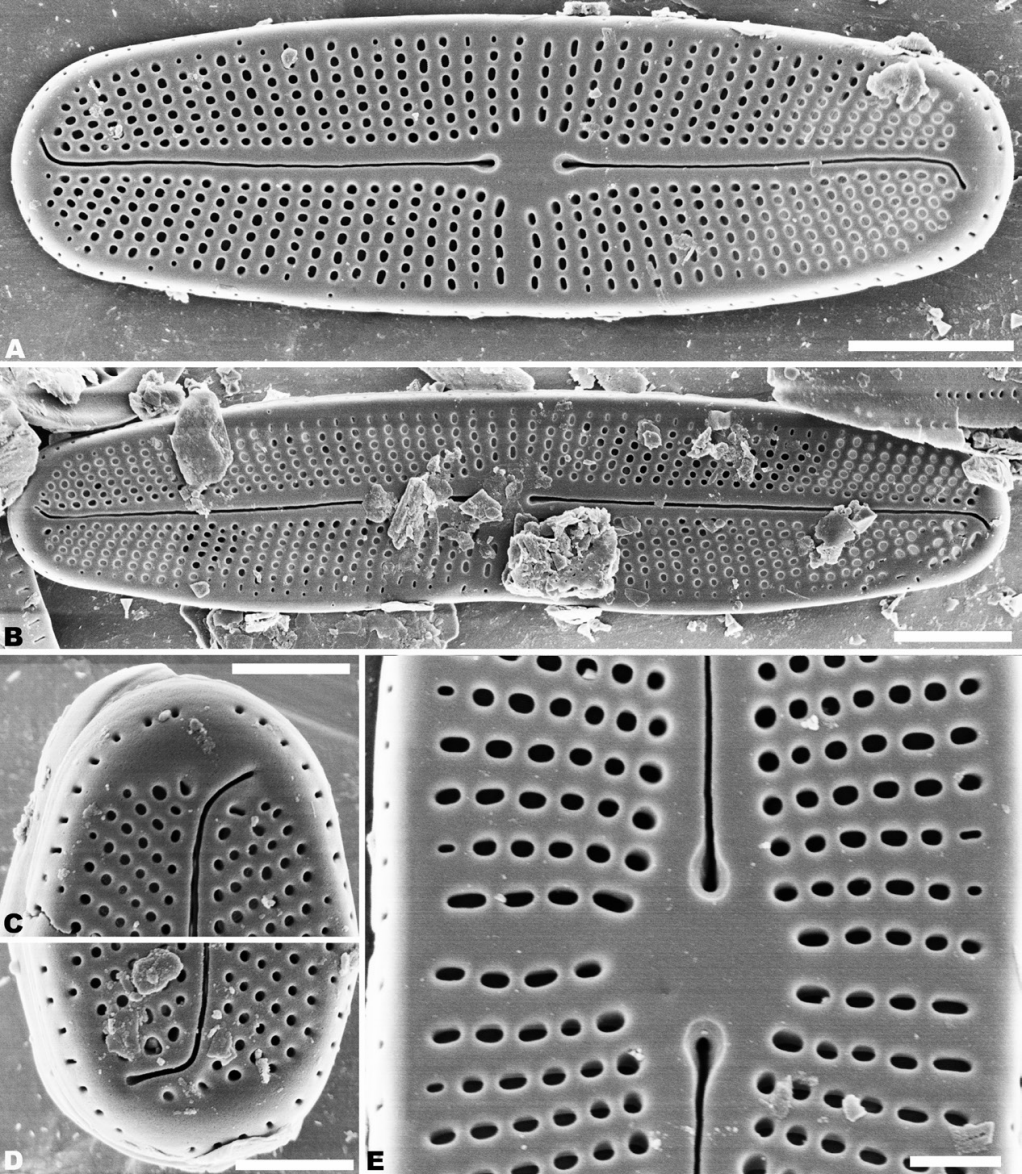
**A–E***Achnanthidiumkangdingnese* sp. nov. SEM external views of raphe valve **A, B** entire raphe valve **C, D** valve apex, showing the distal raphe ends **E** central area of the valve, showing the proximal raphe ends. Scale bars: 2 µm (**A, B**); 1 µm (**C, D**); 0.5 µm (**E**).

**Figure 3.**
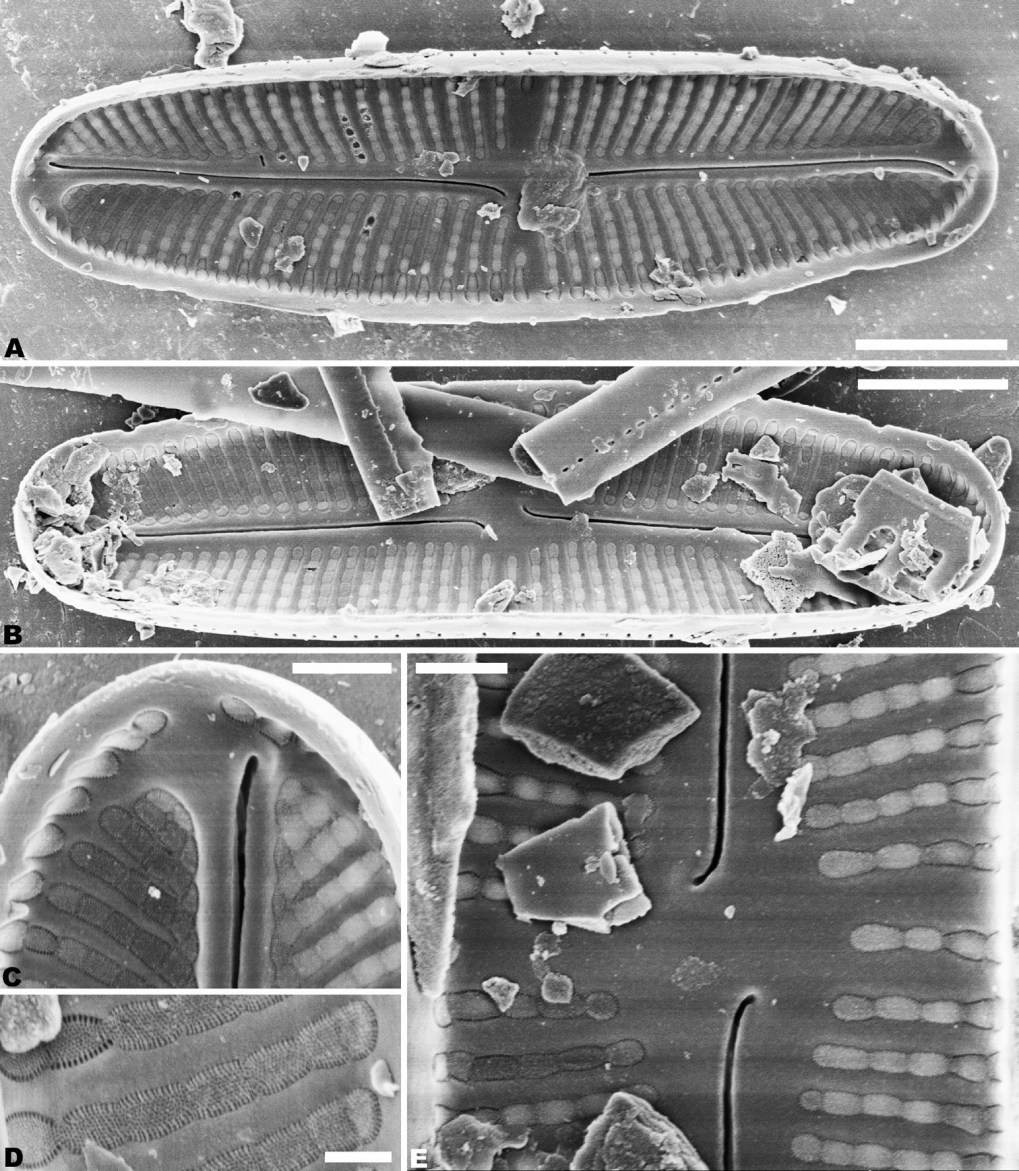
**A–E***Achnanthidiumkangdingnese* sp. nov. SEM internal views of raphe valve **A, B** entire raphe valve **C** valve apex, showing the distal raphe ends **E** central area of the valve, showing the proximal raphe ends **D** internal areola occluded with fine hymenate structures. Scale bars: 2 µm (**A, B**); 1 µm (**C**); 0.5 µm (**E**); 0.2 µm (**D**).

**Figure 4.**
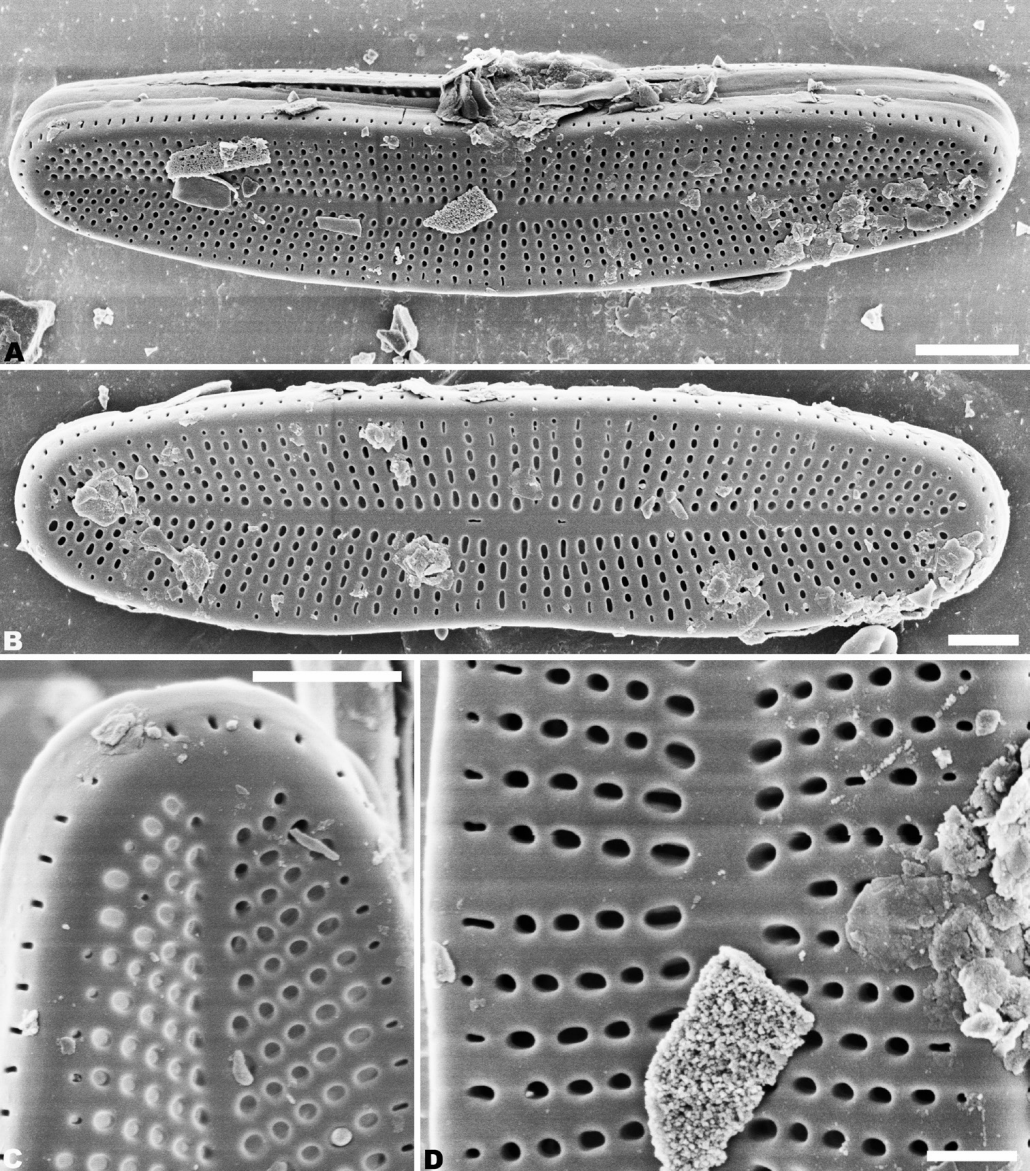
**A–D***Achnanthidiumkangdingnese* sp. nov. SEM external views of rapheless valve **A, B** entire raphe valve **C** valve apex **D** central area of the valve. Scale bars: 2 µm (**A, B**); 1 µm (**C**); 0.5 µm (**D**).

**Figure 5.**
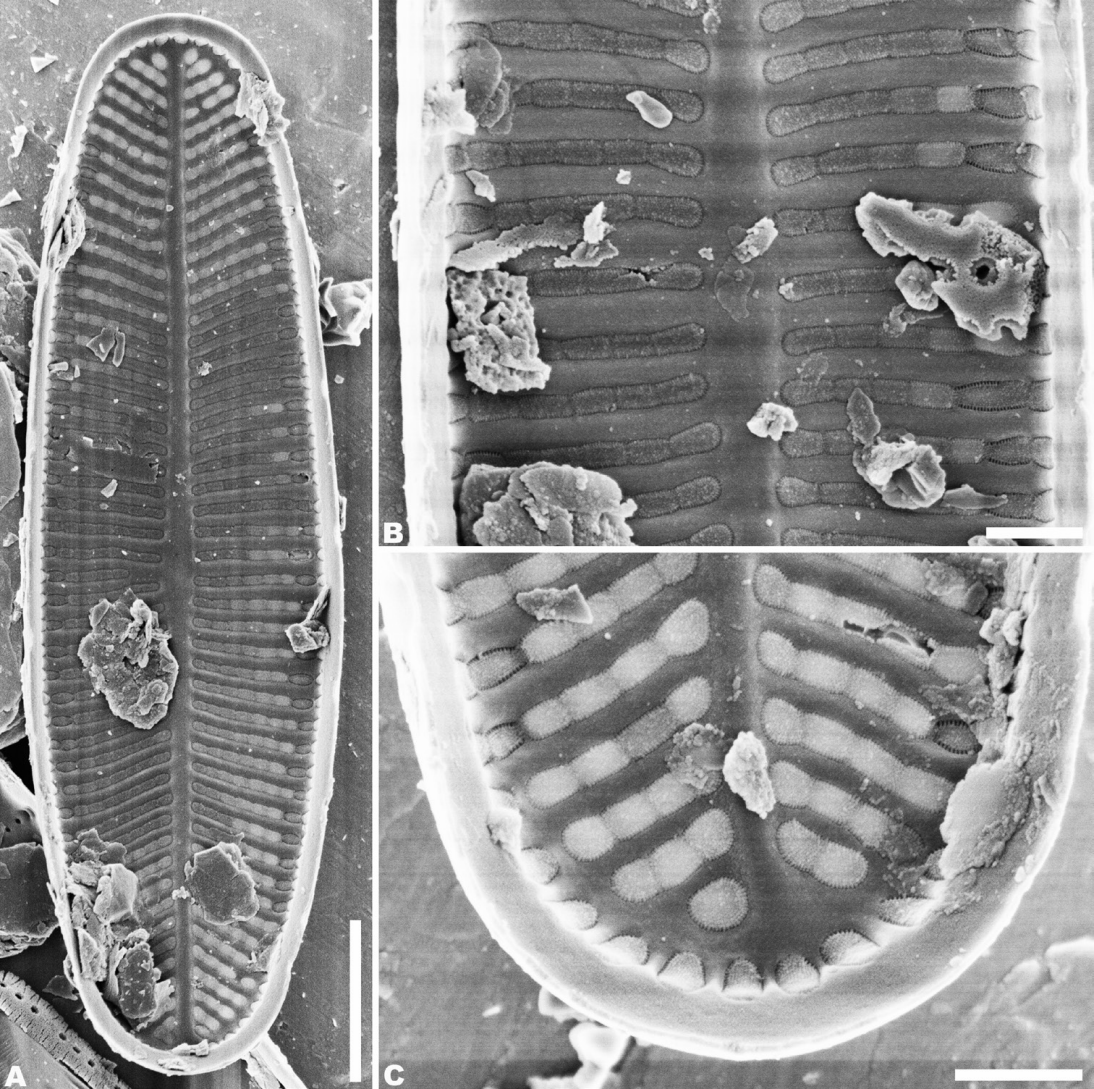
**A–C***Achnanthidiumkangdingnese* sp. nov., SEM internal views of rapheless valve **A** entire raphe valve **B** central area of the valve **C** valve apex. Scale bars: 2 µm (**A**); 0.5 µm (**B, C**).

Figs 1 , 2 , 3 , 4 , 5

#### Description.

LM observations (Fig. 1A–AD), valves are linear in shape, with rounded apices. Some individuals were slightly constricted in the middle. Valve length 10.8–23.5 µm, breadth 3.8–4.0 µm (n = 200). On both valves striae are radiate throughout, and striae count cannot be performed with LM. Raphe valve is concave, with a narrow, linear axial area slightly expanded near the center. The central area is well defined with one or two spaced striae. Rapheless valve is convex, with a narrow linear axial area weakly expanded at the middle portion of the valve. The central area is a small oval or absent.

SEM observations (Figs 2–5), both valves have a narrow hyaline area at the valve face-mantle junction (Figs 2A, B, 4A, B). Raphe valve: Externally, the raphe is filiform and straight (Fig. 2A, B), distal raphe ends are deflected in opposite directions (Fig. 2 A–D), and proximal raphe ends are straight and teardrop-shaped (Fig. 2A, B, E). The number of striae is 34–36 in 10 µm at the middle portion, and 33–38 in 10 µm near the apices (Figs 2A, B; 3A). Areolae are round or oval. The uniseriate striae are composed of 4–7 areolae in the middle portion of the valve (Fig. 2A, B, E), and 1–7 areolae at the apex (Fig. 2 A–D). Valve mantle with a single row of linear areolae extend around the apices with a small interruption at the ends (Fig. 2A, C–E). Internally, the thickening widens at the end (Fig. 3A, C), and the raphe terminates in raised helictoglossae close to the apices (Fig. 3A–C). Proximal raphe ends are distinctly deflected in opposite directions (Fig. 3B, E). Areolae are transapically elongated in throughout valve (Fig. 3C, E). Areolae are occluded by hymene perforated by delicate slits, and each hymen is closely joined with the adjacent hymen (Fig. 3D).

Rapheless valve: the single row of pores on the mantle is continuous (Figs 4A–C, 5A, C). Externally, the axial area is linear and weakly expanded in the central area (Fig. 4A, B). On some valves, there are two slit-like areolae oriented longitudinally in the middle region of the axial area (Fig. 4B). Striae are uniseriate, comprised of 3–6 round or transapically oriented areolae in the central area (Fig. 4A, B, D), and 1–5 round or oblong areolae at the apices (Fig. 4A–C). A row of slit-like areolae is present on the mantle (Fig. 4A, B). Internally, the axial area is slightly raised (Fig. 5A). Areolae are transapically oval in the valve (Fig. 5B, C). The number of striae is 34–38 in 10 µm in the center, and 38–40 in 10 µm near the apices (Figs 4A, B; 5A). Areolae are occluded by hymens perforated by delicate slits, and each hymen is closely joined with an adjacent hymen (Fig. 5B, C).

#### Holotype (designated here).

SHTU! Slide MGC201508036 in Lab of Algae and Environment, College of Life Sciences, Shanghai Normal University, Shanghai, China. Holotype illustrated in Fig. 1D, R.

#### Type locality.

China. Mugecuo lake, Sichuan Province, 30°08'43"N, 101°51'35"E, altitude: 3780 m, *leg. Quanxi Wang in August 2015*.

#### Etymology.

The species so named refers to Kangding County where the holotype was collected.

#### Ecology.

Periphytic diatom samples collected in Mugecuo Lake (MGC201508036), pH 7.8, water temperature 12.5 °C, Conductivity 35 μs.cm^–1^). The sample of this new species occurred at less than 2% relative abundance (total counted 400 valves). There are 5 species that accounted for more than 5% of sample MGC201508036: *Pantocsekiellaocellata* (Pantocsek) K.T. Kiss & E. Ács (Ács et al. 2016) (47.5%), *Brachysirablancheana* H. Lange-Bertalot & G. Moser (Lange-Bertalot and Moser 1994) (9.6%), *Encyonemasilesiacum* (Bleisch) D.G. Mann (Round et al. 1990) (7.3%), *Staurosirapseudoconstruens* (Marciniak) H. Lange-Bertalot (Krammer and Lange-Bertalot 2000) (7.1%), and *Nitzschiafrustulum* (Kützing) A. Grunow (Cleve and Grunow 1880) (5.2%).

#### Distribution.

The new species is known only from the type locality.

## ﻿Discussion

*Achnanthidiumkangdingnese* sp. nov. possesses features characteristic of the genus *Achnanthidium*. These characteristics include a linear shape, with rounded apices, uniseriate striae, transpically-elongated areolae on the both valves, fine raphe, and deflected external distal raphe fissures (Ponader and Potapova 2007). The deflected external distal raphe fissures support its inclusion in the *A.initium*-like group (Karthick et al. 2017).

*Achnanthidiumkangdingnese* can be compared with several conspecific representatives within the genus based on the outline and structure of the valve. Similar species used for comparison include *A.contrarea*, *A.peridotiticum*, *A.indicatrix*, and *A.initium* (Table 1). In terms of features viewed in the LM, the outline of the valves of *A.kangdingnese* are linear with rounded apices, while those of *A.contrarea* were expanded linear to linear-elliptical with broad capitate apices, *A.peridotiticum* and *A.indicatrix* are linear to linear-elliptical with rounded capitate apices. The valves of *A.kangdingnese* are shorter (10.8–23.5 µm) than the valves of *A.contrarea* (15 to 37.0 µm) and *A.indicatrix* (20.0–35.0 µm). The valves of *A.kangdingnese* are wider (3.8–4.0 µm) than the valves of *A.initium* (3.1–3.6 µm), and narrower than *A.contrarea* (6.0–8.0 µm) and *A.indicatrix* (5.0–7.5 µm). *A.kangdingnese* also has a small oval or absent central area, but *A.peridotiticum* and *A.indicatrix* possess a rhombic-shaped central area, *A.contrarea* has a rhombic to rectangular central area, and *A.initium* has an asymmetrical transverse central area. Additionally, the density of striae of *A.kangdingnese* is higher on both valves than in *A.contrarea* (28–32 in 10 µm on both valves), *A.peridotiticum* (~30 in 10 µm on both valves), *A.indicatrix* (24–27 in 10 µm on the raphe valve, 25–30 in 10 µm on the rapheless valve), and *A.initium* (29–34 in 10 µm on the raphe valve, 32–35 in 10 µm on the rapheless valve).

**Table 1.** Comparison of morphological characteristics of *Achnanthidiumkangdingnese* sp. nov. and closely related taxa.

Species/Feature	*A.kangdingnese* sp. nov.	*A.contrarea* (Lange-Bertalot & Steindorf) Lange-Bertalot	*A.peridotiticum* (Moser, Lange-Bertalot & Metzeltin) Lange-Bertalot	*A.indicatrix* (Lange-Bertalot & Steindorf) Lange-Bertalot	*A.initium Karthick, Tayl*or & Hamilton
Valve length (μm)	10.8–23.5	15.0–37.0	15.0–27.0	20.0–35.0	11.0–25.5
Valve width (μm)	3.8–4.0	6.0–8.0	3.5–4.8	5.0–7.5	3.1–3.6
Valve outline	Linear	Expanded linear to linear- elliptical	Linear to linear-elliptical	Expanded linear to linear- elliptical	Linear-lanceolate to lanceolate
Valve apices	Rounded	Broad capitate	Rounded capitate	Rounded capitate	Rounded to weakly rostrate rounded
**Raphe valve**					
Axial area	Linear	Linear, linear-lanceolate	Linear-lanceolate	Linear	Narrow linear
Central area	Small oval or absent	Rhombic to rectangular	Rhombic	Small, rhombic	Asymmetrical transverse
Raphe	Distal fissures deflected to opposite directions	Distal fissures are hooked towards the opposite side	Distal fissures are strongly hooked towards the opposite side	Distal fissures are strongly hooked towards the opposite side	Distal fissures are strongly hooked towards the opposite side
Density of striae (10 μm)	34–36 (middle), 33–38 (apices)	28–32	~30	24–27	29–34
Number of areolae per stria	4–7 (middle), 1–7 (apices)	1–5 (middle), 1–4 (apices)	No data	5–6 (middle), 1–5 (apices)	2–5 (middle), 1–4 (apices)
**Rapheless valve**					
Axial area	Narrow, linear	Lanceolate	Linear	Linear	Narrow linear
Central area	Absent	Absent	Absent	Absent	Absent or weakly elliptical
Density of striae (10 μm)	34–38 (middle), 38–40 (apices)	28–32	~30	25–30	32–35
Number of areolae per stria	3–6 (middle), 1–5 (apices)	1–2 (middle), 1–3 (apices)	No data	No data	4–5 (middle), 1–3 (apices)
References	Current study	Moser et al. (1998)	Moser et al. (1998)	Moser et al. (1998)	Karthick et al (2017)

*Achnanthidiumkangdingnese* is easily separated from *A.minutissimum* complex and *A.pyrenaicum* complex species in this genus by having external distal raphe ends curved in opposite directions of the valve. In contrast to other *Achnanthidium* species, in an internal view, the areolae of *A.kangdingnese* are occluded by hymens, and each hymen closely joins with the adjacent hymen on the both valves (Figs 3C–E, 5A–C).

*Achnanthidiumkangdingnese* has only been found on stones in Mugecuo Lake. This lake has a slightly alkaline pH (7.8) and low conductivity (35 μs.cm^–1^). Among the four samples taken from Mugecuo Lake, *A.kangdingnese* was found, in low numbers, only in one sample. At the type locality, other monoraphid species co-occur with these new species. The co-occurring monoraphid taxa include *A.pyrenaicum* (Hustedt) P. Kobayasi (Kobayashi 1997), *A.rivulare* M.G. Potapova and K.C. Ponader (Potapova and Ponader 2004), *A.minutissimum* (Kützing) D.B. Czarnecki (Czarnecki 1994), and *Eucocconeislaevis* (Østrup) H. Lange-Bertalot (Lange-Bertalot and Genkal 1999). Further studies are needed to clarify the relationship between diatom diversity and ecology in this region.

## Supplementary Material

XML Treatment for Achnanthidium kangdingnese

## References

[B1] Ács E Ari E Duleba M Dressler M Genkal SI Jakó E Rimet F Ector L Kiss KT (2016) *Pantocsekiella*, a new centric diatom genus based on morphological and genetic studies. Fottea, Olomouc 16(1): 56–78. 10.5507/fot.2015.028

[B2] Bory de Saint-Vincent JBGN (1822) Achnanthe. *Achnanthes*. Dictionnaire Classique d’Histoire Naturelle 1: 79–80.

[B3] Chen Y Lei B Luo CD Wang HY Ma D Liu L (2013) Study on soil organic matter and predict its storage of abies faxoniana forest in Mugecuo, Kangding. Journal of Soil and Water Conservation 27(4): 252–257.

[B4] Cleve PT Grunow A (1880) Beiträge zur Kenntniss der arctischen Diatomeen. Kongliga Svenska Vetenskaps-Akademiens Handlingar 17(2): 121.

[B5] Czarnecki DB (1994) The freshwater diatoms culture collection at Loras College, Dubuque, Iowa. In: Kockiolek JP (Ed.) Proceedings of the 11th International Diatom Symposium.Memoirs of the California Academy of Sciences 17: 155–174.

[B6] Ge DY Zhang SM Liu HY Liu Y Kociolek PJ Zhu H Liu GX Fan YW (2022) One new species of *Achnanthidium* Kützing (Bacillariophyta, Achnanthidiaceae) from Tibet, China. Phytotaxa 538(4): 268–280. 10.11646/phytotaxa.538.4.1

[B7] Hustedt F (1922) Bacillariales aus Innerasien. Gesammelt von Dr. Sven Hedin. In: Hedin S (Ed.) Southern Tibet, discoveries in former times compared with my own researches in 1906–1908.Lithographic Institute of the General Staff of the Swedish Army, Stockholm 6(3), 107–152. 10.5962/bhl.title.64226

[B8] Jao C (1964) Some fresh-water algae from southern Tibet. Oceanologia et Limnologia Sinica 6(2): 169–192. https://doi.org/CNKI:SUN:HYFZ.0.1964-02-003

[B9] Jao C Zhu H Lee Y (1974) The fresh-water algae from Mount Qomolangma district (in Tibet). Report of the Scientific Survey of Mount Qomolangma district 1966–1968: 92–126.

[B10] Karthick B Taylor JC Hamilton PB (2017) Two new species of *Achnanthidium* Kützing (Bacillariophyceae) from Kolli Hills, Eastern Ghats, India. Fottea 17(1): 65–77. 10.5507/fot.2016.020

[B11] Kobayashi H (1997) Comparative studies among four linear-lanceolate *Achnanthidium* species (Bacillariophyceae) with curved terminal raphe endings. Nova Hedwigia 65(1–4): 147–164. 10.1127/nova.hedwigia/65/1997/147

[B12] Kociolek JP Balasubramanian K Blanco S Coste M Ector L Liu Y Kulikovskiy M Lundholm N Ludwig T Potapova M Rimet F Sabbe K Sala S Sar E Taylor J Van de Vijver B Wetzel CE Williams DM Witkowski A Witkowski J (2018) In DiatomBase. http://www.diatombase.org [Accessed: 2018–03–15]

[B13] Kociolek JP You Q Liu Q Liu Y Wang Q (2020) Continental diatom biodiversity discovery and description in China: 1848 through 2019. PhytoKeys 160: 45–97. 10.3897/phytokeys.160.54193 32982550 PMC7492188

[B14] Krammer K Lange-Bertalot H (2000) Bacillariophyceae, 3. Teil: Centrales, Fragilariaceae, Eunotiaceae. Spektrum Akademischer Verlag, Heidelberg, 578 pp.

[B15] Kulikovskiy MS Maltsev YI Glushchenko AM Kuznetsova IV Kapustin DA Lange-Bertalot H Genkal SI Kociolek JP (2020) *Gogorevia*, a new monoraphid diatom genus for *Achnanthesexigua* and allied taxa (Achnanthidiaceae) described on the basis of an integrated molecular and morphological approach. Journal of Phycology 56(6): 1601–1613. 10.1111/jpy.13064 32871027

[B16] Kützing FT (1844) Die Kieselschaligen Bacillarien oder Diatomeen. W. Köhne, Nordhausen, 152 pp. 10.5962/bhl.title.64360

[B17] Lange-Bertalot H Genkal SI (1999) Diatoms from Siberia I - Islands in the Arctic Ocean (Yugorsky-Shar Strait) Diatomeen aus Siberien. I. Insel im Arktischen Ozean (Yugorsky-Shar Strait). Iconographia Diatomologica 6: 271.

[B18] Lange-Bertalot H Moser G (1994) *Brachysira*. Monographie der Gattung und *Naviculadicta* nov. gen. Biblioteca Diatomologica 29: 212.

[B19] Liu B Blanco S Long H Jingjing XU Jiang X (2016) *Achnanthidiumsinense* sp. nov. (Bacillariophyta) from the Wuling Mountains Area, China. Phytotaxa 284(3): 194–202. 10.11646/phytotaxa.284.3.4

[B20] Liu Y Tan X Kociolek JP Kulikovskiy M Lu XX Fan YW (2021) One new species of *Achnanthidium* Kützing (Bacillariophyta, Achnanthidiaceae) from the upper Han River, China. Phytotaxa 516(2): 187–194. 10.11646/phytotaxa.516.2.6

[B21] Miao M Li Z Hwang EA Kim HK Lee H Kim BH (2020) Two New Benthic Diatoms of the Genus *Achnanthidium* (Bacillariophyceae) from the Hangang River, Korea. Diversity (Basel) 12(7): 285. 10.3390/d12070285

[B22] Moser G Lange-Bertalot H Metzeltin D (1998) Insel der Endemiten Geobotanisches Phänomen Ne-ukaledonien (Island of endemics New Caledonia -a geobotanical phenomenon). Bibliotheca Diatomologica 38: 464.

[B23] Parr JF Taffs KH Lane CM (2004) A microwave digestion technique for the extraction of fossil diatoms from coastal lake and swamp sediments. Journal of Paleolimnology 31(3): 383–390. 10.1023/B:JOPL.0000021857.32734.c6

[B24] Pérès F Cohu RL Delmont D (2014) *Achnanthidiumbarbei* sp. nov. and *Achnanthidiumcostei* sp. nov., two new diatom species from French rivers. Diatom Research 29(4): 387–397. 10.1080/0269249X.2014.890956

[B25] Ponader KC Potapova MG (2007) Diatoms from the genus *Achnanthidium* in flowing waters of the Appalachian Mountains (North America): Ecology, distribution and taxonomic notes. Limnologica 37(3): 227–241. 10.1016/j.limno.2007.01.004

[B26] Potapova MG Ponader KC (2004) Two common North American diatoms, *Achnanthidiumrivulare* sp. nov. and *A.deflexum* (Reimer) Kingston: Morphology, ecology and comparison with related species. Diatom Research 19(1): 33–57. 10.1080/0269249X.2004.9705606

[B27] Qi YZ Xie SQ (1984) The diatom in moss swamp from Hubei Shennongjia. Jinan Yili Xuebao 1984(3): 86–92.

[B28] Round FE Bukhtiyarova L (1996) Four new genera based on Achnanthes (Achnanthidium) together with re-definition of *Achnanthidium*. Diatom Research 11(2): 345–361. 10.1080/0269249X.1996.9705389

[B29] Round FE Crawford RM Mann DG (1990) The Diatoms. Biology and morphology of the genera. Cambridge University Press, Cambridge, 747 pp.

[B30] Tseplik ND Maltsev YI Glushchenko AM Kuznetsova IV Genkal SI Kociolek JP Kulikovskiy MS (2021) *Achnanthidiumtinea* sp. nov.-a new monoraphid diatom (Bacillariophyceae) species, described on the basis of molecular and morphological approaches. PhytoKeys 174: 147–163. 10.3897/phytokeys.174.60337 33776528 PMC7979678

[B31] You QM Cao Y Yu P Kociolek JP Zhang LX Wu B Lowe R Wang QX (2019) Three new subaerial *Achnanthidium* (Bacillariophyta) species from a karst landform in the Guizhou Province, China. Fottea 19(2): 138–150. 10.5507/fot.2019.005

[B32] You QM Zhao K Wang YL Yu P Kociolek JP Pang WT Wang QX (2021) Four new species of monoraphid diatoms from Western Sichuan Plateau in China. Phytotaxa 479(3): 257–274. 10.11646/phytotaxa.479.3.3

[B33] Yu P Kociolek JP You QM Wang QX (2018) *Achnanthidiumlongissima* sp. nov. (Bacillariophyta), a new diatom species from Jiuzhai Valley, Southwestern China. Diatom Research 33(3): 339–348. 10.1080/0269249X.2018.1545704

[B34] Yu P You Q Kociolek JP Wang Q (2019a) Three new freshwater species of the genus *Achnanthidium* (Bacillariophyta, Achnanthidiaceae) from Taiping Lake, China. Fottea 19(1): 33–49. 10.5507/fot.2018.015

[B35] Yu P You QM Pang WT Cao Y Wang QX (2019b) Five new Achnanthidiaceae species (Bacillariophyta) from Jiuzhai Valley, Sichuan Province, Southwestern China. Phytotaxa 405(3): 147–170. 10.11646/phytotaxa.405.3.5

[B36] Yu P You Q Pang W Wang Q (2022) Two new freshwater species of the genus *Achnanthidium* (Bacillariophyta, Achnanthidiaceae) from Qingxi River, China. PhytoKeys 191: 11–28. 10.3897/phytokeys.191.78489 PMC8917116 35437388

[B37] Zhu HZ Chen JY (1994) Study on the diatoms of the Wuling Mountain Region. Compilation of reports on the survey of algal resources, 405 pp.

[B38] Zhu HZ Chen JY (1996) New taxa of diatom (Bacillariophyta) from Xizang (Tibet). (II). Zhiwu Fenlei Xuebao 34(1): 102–104. https://doi.org/CNKI:SUN:ZWFX.0.1996-01-011

